# Plasma p‐tau181 as a Marker of Conversion to Alzheimer's Disease Dementia and Worsening in Cognitive Functions in Subjective Cognitive Decline and Mild Cognitive Impairment: A Longitudinal Study

**DOI:** 10.1002/acn3.70190

**Published:** 2025-09-10

**Authors:** Giulia Giacomucci, Assunta Ingannato, Chiara Crucitti, Silvia Bagnoli, Elisa Marcantelli, Sonia Padiglioni, Valentina Moschini, Carmen Morinelli, Laura Falsini, Sandro Sorbi, Valentina Berti, Benedetta Nacmias, Valentina Bessi

**Affiliations:** ^1^ Department of Neuroscience, Psychology, Drug Research and Child Health University of Florence Florence Italy; ^2^ Research and Innovation Centre for Dementia‐CRIDEM, AOU Careggi Florence Italy; ^3^ Regional Referral Centre for Relational Criticalities – Tuscany Region Florence Italy; ^4^ SOD Neurologia, Dipartimento Neuromuscolo‐Scheletrico e degli Organi di Senso, AOU Careggi Florence Italy; ^5^ University of Florence Florence Italy; ^6^ IRCCS Fondazione Don Carlo Gnocchi Florence Italy; ^7^ Department of Biomedical, Experimental and Clinical Sciences “Mario Serio” University of Florence Florence Italy; ^8^ Nuclear Medicine Unit Azienda Ospedaliero‐Universitaria Careggi Florence Italy

**Keywords:** Alzheimer's disease, cognitive worsening, mild cognitive impairment, plasma biomarkers, plasma p‐tau181, subjective cognitive decline

## Abstract

**Background:**

Plasma p‐tau181 has proven to be a promising diagnostic and prognostic tool in the earliest phases of Alzheimer's disease (AD). We aimed to evaluate the prognostic role of p‐tau181 in predicting conversion to AD dementia and worsening in cognition in mild cognitive impairment (MCI) and subjective cognitive decline (SCD).

**Methods:**

We consecutively enrolled 163 patients (50 SCD, 70 MCI, and 43 ad‐demented (AD‐d)), who underwent plasma p‐tau181 analysis with the Simoa assay. Patients were classified according to the Revised Criteria of the Alzheimer's Association Workgroup as Core1+ or Core1− (based on amyloid‐PET, CSF Aβ42/Aβ40, CSF p‐tau181/Aβ42).

**Results:**

Plasma p‐tau181 levels were significantly influenced by Core1 status (*B* = 1.41, *p* < 0.001) and clinical diagnosis (*B* = 0.63, *p* < 0.001). Plasma p‐tau181 was highly accurate in discriminating between Core1+ and Core1− patients (AUC = 0.88 [95% CI 83.00–94.00]) with a cut‐off value of 2.25 pg/mL presenting good accuracy (85.90%), specificity (74.58%), and excellent sensitivity (92.78%). Classifying patients according to p‐tau181 cut‐off, we found that p‐tau181+ patients showed an increased risk of converting to AD dementia (HR = 11.65, *p* = 0.018). Moreover, SCD p‐tau181+ worsened over time in tasks assessing long‐term verbal (*p* = 0.012) and spatial memory (*p* = 0.009).

**Conclusions:**

Plasma p‐tau181 is not only a good diagnostic marker for AD pathology, but it also plays a role as a predictor of both conversion to AD dementia and of worsening of cognitive performance since the earliest phase of AD.

## 1 Introduction

The detection of Alzheimer's disease (AD) before the damage due to neurodegeneration has taken its toll assumes an even greater importance when considering the recent approval of targeted disease‐modifying treatments, whose practical application in the management of AD is expected to become a reality in the near future [1].

Specifically, cognitively undamaged and mildly cognitively impaired individuals represent the ideal group in which to efficaciously intervene to prevent further injury to neural cells; this includes both patients with mild cognitive impairment (MCI) [2] and subjective cognitive decline (SCD), a condition defined as the detection of a deterioration compared to previous cognitive capabilities without an objective validation [3].

However, the etiology underlying SCD includes such heterogeneous pathological causes that identifying subjects who are actually in the process of converting to AD dementia is essential. Similarly, MCI also represents a heterogeneous condition, with patients progressing to different types of dementia, while others remain stable or even revert to normal cognition [2, 4]. For this reason, several efforts have been made to stratify both SCD and MCI patients according to their risk of progression—towards objective cognitive impairment in SCD, and towards dementia in MCI [5, 6, 7].

Plasma biomarkers currently represent the most promising resource in the field of AD, also considering the recent revision of diagnostic criteria by the Alzheimer's Association Workgroup, which proposed their use for diagnostic purpose [8]. Although phosphorylated tau isoform 217 (p‐tau217) has recently emerged as the most promising biomarker for AD diagnosis due to its higher specificity and sensitivity, plasma p‐tau181 remains a valuable and widely studied marker, identifying individuals at risk of developing AD dementia even when clinical symptoms are not yet so prominent [9, 10].

According to existing literature [11, 12, 13], plasma p‐tau181 levels differed among patients depending only on the underlying AD pathology, thus effectively differentiating between carriers and non‐carriers of AD pathology even in the early stages of the disease, particularly in the SCD population [14].

On the other hand, growing evidence supports its role as a prognostic biomarker, capable of predicting not only the presence of AD pathology but also the clinical evolution of the disease, such as conversion to AD dementia [9, 12]. Moreover, plasma p‐tau181 has also shown promising associations with longitudinal deterioration in cognitive performance [15].

Considering these premises, plasma p‐tau181 might not only serve as a biological indicator of underlying AD pathology, but also act as an early warning signal for upcoming cognitive decline, even in individuals who are still objectively cognitively unimpaired. From a practical standpoint, the clarification of prognostic role of p‐tau181 might be extremely useful in this translation of plasma biomarkers from research setting to clinical practice. For these reasons, the aims of our study were:

1. To assess plasma p‐tau181 cut‐off value to detect AD in SCD and MCI adopting the recently revised criteria drawn up by the Alzheimer's Association Workgroup [8];
2. To evaluate the prognostic role of plasma p‐tau181 as an effective, accurate and noninvasive instrument to intercept SCD and MCI patients who will convert to AD dementia [16];
3. To evaluate whether higher levels of plasma p‐tau181 at baseline could be indicative of the deterioration of cognitive performance [15, 17] in SCD and MCI patients.

## 2 Materials and Methods

### 2.1 Participants

Between July 2018 and September 2024, we consecutively enrolled and collected plasma sample of 163 patients (50 SCD, 70 MCI and 43 ad demented) referred to the Centre for Alzheimer's disease and Adult Cognitive Disorders of Careggi Hospital in Florence.

Patients met the following inclusion criteria:

1. receiving a clinical diagnosis of AD dementia according to the NIA‐AA criteria, including the atypical variant [18],
2. receiving a clinical diagnosis of MCI according to NIA‐AA criteria [2],
3. receiving a clinical diagnosis of SCD according to SCD‐I criteria [3].

At baseline, patients underwent comprehensive family and clinical history, neurological examination and extensive neuropsychological investigation (described in detail elsewhere [19]), blood collection for measurement of plasma p‐tau181 concentration and genetic analysis. SCD and MCI patients repeated neuropsychological investigation after two years (T1) from baseline evaluation (T0).

Renal function was categorized as either impaired or not impaired based on estimated glomerular filtration rate (eGFR; considered impaired if < 60 mL/min/1.73 m^2^).

A total of 145 patients (36 SCD, 66 MCI, 43 ad‐d) underwent CSF collection for amyloid‐β (Aβ)42, Aβ42/Aβ40, t‐tau, p‐tau, and p‐tau/Aβ42. Normal values for CSF biomarkers were: Aβ42 > 670 pg/mL, Aβ42/Aβ40 > 0.062, t‐tau < 400 pg/mL, p‐tau < 60 pg/mL, p‐tau/Aβ42 < 0.068. Forty‐four patients (24 SCD, 14 MCI and 6 ad‐d) underwent amyloid‐PET scans. Both CSF collection and amyloid‐PET scans were performed in 33 patients (17 SCD, 10 MCI and 6 ad‐d). *APOE* genotyping was available for 152 patients (47 SCD, 66 MCI, 39 ad‐d). Methods used for CSF collection, *APOE* genotyping, CSF analysis, brain amyloid‐PET acquisition and rating are described in further detail elsewhere [14, 20, 21, 22].

### 2.2 Classification of Patients According to the Revised Criteria of Alzheimer's Association Workgroup

Based on biomarker results, patients were classified according to the Revised Criteria of Alzheimer's Association Workgroup. Patients were rated as Core1+ in case of abnormality on at least one of specific Core1 biomarkers (amyloid PET, CSF Aβ42/Aβ40, CSF p‐tau 181/Aβ42), and as Core1− in case of normal Core1 biomarkers [8]. Patients were further classified according to both diagnosis (SCD, MCI, AD‐d) and Core1 biomarkers' results as follows: SCD Core1−, SCD Core1+, MCI Core1−, MCI Core1+, AD‐d.

### 2.3 Plasma p‐tau181

Blood samples were collected by venipuncture into standard polypropylene EDTA test tubes (Sarstedt, Nümbrecht, Germany) at 8:00 AM, with participants in a fasting state. Plasma was isolated from peripheral blood samples within 2 h of collection. Blood samples were centrifugated at 1300 rcf for 10 min, and plasma was stored at −80°C until tested. The Simoa Human p‐tau181 Advantage V2 kit (item #103714, provided by Quanterix Corp.—Billerica, MA, USA) was used for the quantitative determination of p‐tau181 in plasma samples on the automated Simoa SR‐X instrument (Quanterix Corp.—GBIO, Hangzhou, China). The kit's Analytical Lower Limit of Quantification (LLOQ) value was 0.085 pg/mL, while the kit's Limit of Detection (LOD) was 0.041 pg/mL (range 0.018–0.060 pg/mL). For the run setup, 7 calibrators and 2 controls, provided by Quanterix, were required for the analysis. Calibrators were used to set a calibration curve of serial measurements; controls were the lower and higher target concentrations. Plasma samples and controls were diluted 4×. Calibrators, controls, and samples were run in duplicate, detected on a single run basis [23].

### 2.4 Statistical Analysis

All statistical analyses were performed using IBM SPSS Statistics software version 25 (SPSS Inc., Chicago, Illinois), Jamovi (Jamovi version 2.3) and the computing environment R4.2.3 (R Foundation for Statistical Computing, Vienna, 20, 13). All *p* values were two‐tailed, with the significance level set at *p* = 0.05. Descriptive statistics included means and standard deviations for continuous variables, and frequencies, percentages, and 95% confidence intervals (CIs) for categorical variables. The Shapiro–Wilk test was used to explore data distribution. Depending on data distribution, between‐group comparisons were conducted using either *t*‐tests or non‐parametric Mann–Whitney *U* tests. Correlations between numeric variables were assessed using Pearson's correlation coefficient or Spearman's *ρ* (rho) as appropriate. Categorical variables were compared with the Chi‐square test. Differences in continuous variables across multiple groups were analyzed using one‐way ANOVA followed by Bonferroni post hoc tests. Effect sizes were calculated using partial *η* ^2^, Cohen's *d*, rank biserial correlation coefficient (*r*), and Cramer's *V*. To adjust for possible confounding factors, ANCOVA was applied. Multiple linear regression analyses were performed to identify variables independently associated with plasma p‐tau181 levels. Receiver Operating Characteristic (ROC) analyses evaluated the ability of plasma p‐tau181 to distinguish between Core1+ and Core1− patients. The optimal cut‐off value for plasma p‐tau181 was determined by maximizing the sum of sensitivity and specificity, and diagnostic accuracy was assessed through positive and negative predictive values (PPV and NPV).

Participants were stratified into plasma p‐tau181 positive or negative groups based on this cut‐off. To assess the risk of conversion to AD dementia, we included SCD and MCI patients who converted to AD dementia or those who remained stable with a follow‐up time > 3 years. Survival analyses were conducted using Kaplan–Meier estimates and Cox proportional hazards regression, with time defined as the interval (in years) from baseline to diagnosis or last follow‐up. Kaplan–Meier survival curves were compared using the log‐rank test. Logistic regression analysis was run to define which variables might influence conversion to AD dementia. Mixed‐effects linear models were used to examine the longitudinal changes in neuropsychological test scores as a function of diagnostic group (SCD and MCI) and plasma p‐tau181 status.

## 3 Results

### 3.1 Distribution of Plasma p‐tau181 Across Clinically Defined Groups

Demographic features and differences among diagnostic groups are summarized in Table 1. Impaired renal function was found in two AD‐d and one MCI patient, with no differences in terms of the proportion of renal impairment among the SCD, MCI, and AD‐d groups. Plasma p‐tau181 concentration was correlated with age at plasma collection (Spearman's *ρ* 0.326, *p* < 0.001) and age at onset (Spearman's *ρ* 0.433, *p* < 0.001). Plasma p‐tau181 levels were different among SCD, MCI, and AD‐d patients (*F* [2160] = 30.62, *p* < 0.001, partial *η* ^2^ = 0.277), also after correcting for age (*F* [3159] = 24.80, *p* < 0.001, partial *η* ^2^ = 0.259). Plasma p‐tau181 levels were higher in AD‐d than in MCI (*p* < 0.001) and than in SCD (*p* < 0.001); moreover, MCI patients showed higher plasma p‐tau181 concentration than SCD subgroups (*p* = 0.006) (Table 1). AD biomarkers positivity was described in Table S1.

**TABLE 1:** Demographic features of subjective cognitive decline (SCD), mild cognitive impairment (MCI) and Alzheimer's disease dementia (AD‐d) groups.

	SCD	MCI	AD‐d
	N° 50	N° 70	N° 43
Age at onset in years	57.56 (±8.95) ^a,b^	66.00 (±9.33) ^a^	67.19 (±6.94) ^b^
Age at plasma collection	66.80 (±8.07) ^c^	70.78 (±7.83) ^c^	70.56 (±6.86)
Family history of AD	81.60% ^d^	66.20%	56.10% ^d^
Sex (M–F)	13–37 ^e^	36–34 ^e^	19–24
Years of education	12.98 (±4.05) ^f^	12.23 (±4.20) ^g^	9.82 (±4.63) ^f,g^
MMSE	28.47 (±1.53) ^h^	27.46 (±2.28) ^i^	21.00 (±5.66) ^h,i^
*APOE* ɛ4+	34.00%	40.90%	56.40%
Impaired renal function	0	1 (1.43%)	2 (4.65%)
Plasma p‐tau181 (pg/mL)	2.15 (±0.90) ^j,k^	2.95 (±1.53) ^k,l^	4.36 (±1.53) ^j,l^

*Note:* Values are reported as mean and standard deviation or frequencies or percentages for continuous variables and categorical variables respectively. Statistically significantly different values between the groups are reported as underlined character. Statistical significance: *p* < 0.05. ^a^ *p* < 0.001; ^b^ *p* < 0.001; ^c^ *p* = 0.017; ^d^ *χ* ^2^ 6.93, *p* = 0.011; ^e^ *χ* ^2^ 7.80, *p* = 0.008; ^f^ *p* = 0.002; ^g^ *p* = 0.015; ^h^ *p* < 0.001; ^i^ *p* < 0.001; ^j^ *p* = 0.006; ^k^ *p* < 0.001; ^l^ *p* < 0.001.

Abbreviations: F, females; M, males; MMSE, mini mental state examination.

### 3.2 Distribution of Plasma p‐tau181 Across Clinically and Biologically Defined Groups

**FIGURE 1:**
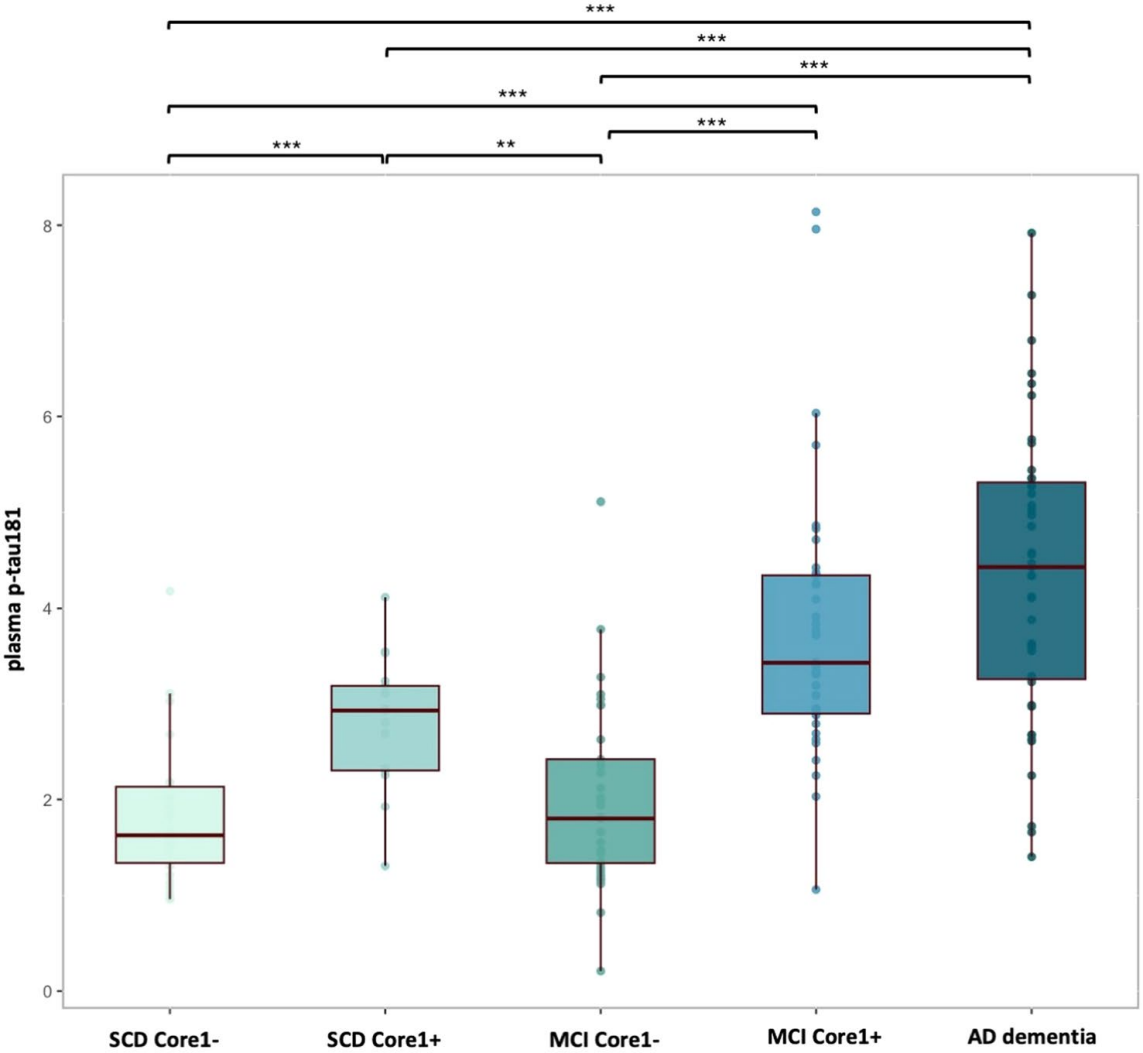
Distribution of plasma p‐tau181 across clinically and biologically defined groups. Values quoted in the *y*‐axis indicate plasma p‐tau181 levels (pg/mL). Horizontal bars indicate significant differences between groups. **p < 0.01; ***p < 0.001.

Patients were classified according to both diagnosis and Core1 biomarkers: SCD Core1− (n. 26), SCD Core1+ (n. 17), MCI Core1− (n. 33), MCI Core1+ (n. 37), AD‐d (n. 43, all Core1+). Demographic variables are described in Table 2. Plasma p‐tau181 levels were different among the groups (*F* [4151] = 28.88, *p* < 0.001, partial *η* ^2^ = 0.433), also after adjusting for age (*F* [5150] = 30.30, *p* < 0.001, partial *η* ^2^ = 0.400). Indeed, plasma p‐tau181 levels were significantly higher in SCD Core1+ than in SCD Core1− (2.79 ± 0.68 vs. 1.82 ± 0.775, *p* < 0.001, Cohen's *d* = 0.70) and in MCI Core1+ than in MCI Core1− (3.80 ± 1.44 vs. 1.98 ± 0.97, *p* < 0.001, Cohen's *d* = 1.48). AD‐d patients showed the highest plasma p‐tau181 levels (4.36 ± 1.52). No differences between AD‐d and MCI Core1+ were detected (Figure 1).

**TABLE 2:** Demographic features of diagnostic and biomarker groups.

	SCD Core1−	SCD Core1+	MCI Core1−	MCI Core1+	AD‐d
	N° 26	N° 17	N° 33	N° 37	N° 43
Age at onset in years	56.34 (±7.36) ^a,b^	58.29 (±11.69) ^c,d^	62.69 (±10.90) ^e^	68.94 (±6.52) ^a,c^	67.19 (±6.94) ^b,d,e^
Age at plasma collection	64.69 (±7.39) ^f,g^	69.30 (±9.23)	68.63 (±8.98)	72.71 (±6.14) ^f^	70.54 (±6.87) ^g^
Family history of AD	72.00%	88.20% ^h^	62.50%	69.40%	56.10% ^h^
Sex (M–F)	6–20 ^i^	6–11	16–17	20–17 ^i^	19–24
Years of education	12.36 (±3.92)	13.17 (±4.68)	13.00 (±4.05) ^j^	11.54 (±4.27)	9.83 (±4.63) ^j^
MMSE	28.79 (±1.61) ^k^	28.29 (±1.40) ^l^	27.84 (±2.16) ^m^	27.06 (±2.39) ^n^	21.00 (±5.66) ^k.l,m,n^
*APOE* ɛ4+	33.30% ^o^	35.30%^l,m^	13.30% ^p,q^	63.90% ^o,p^	56.40% ^q^
Impaired renal function	0	0	1 (3.03%)	0	2 (2.90%)
Plasma p‐tau181 (pg/mL)	1.82 (±0.75) ^r,s,t^	2.79 (±0.67) ^r,u,w^	1.99 (±0.97) ^u,x,y^	3.80 (±1.44) ^s,x^	4.36 (±1.52) ^t,w,y^

*Note:* Values are reported as mean and standard deviation or frequencies or percentages for continuous variables and categorical variables respectively. Statistically significantly different values between the groups are reported as underlined character. Statistical significance: *p* < 0.05. ^a^ *p* < 0.001; ^b^ *p* < 0.001; ^c^ *p* < 0.001; ^d^ *p* = 0.004; ^e^ *p* = 0.026; ^f^ *p* < 0.001; ^g^ *p* = 0.022; ^h^ *χ* ^2^ 5.49, *p* = 0.032; ^i^ *χ* ^2^ 6.04, *p* = 0.020; ^j^ *p* = 0.023; ^k^ *p* < 0.001; ^l^ *p* < 0.001; ^m^ *p* < 0.001; ^n^ *p* < 0.001; ^o^ *χ* ^2^ 5.38, *p* = 0.034; ^p^ *χ* ^2^ 17.30, *p* < 0.001; ^q^ *χ* ^2^ 13.40, *p* < 0.001; ^r^ *p* < 0.001; ^s^ *p* < 0.001; ^t^ *p* < 0.001; ^u^ *p* = 0.002; ^w^ *p* < 0.001; ^x^ *p* < 0.001; ^y^ *p* < 0.001.

Abbreviations: F, females; M, males; MMSE, mini mental state examination.

To analyze which factors might influence plasma p‐tau181 levels, we ran a multiple backward regression analysis, considering diagnosis (SCD, MCI, AD dementia), age at plasma collection, Core1 status, impaired renal function, and *APOE* genotypes as covariates. The multiple regression model significantly predicted plasma p‐tau181 levels (*F* [6139] = 22.34, *p* < 0.001, adj. *R* ^2^ = 0.469). Among the covariates, diagnostic category (*B* = 0.63, *p* < 0.001) and Core1 status (*B* = 1.41, *p* < 0.001) added statistical significance to the prediction.

### 3.3 Plasma p‐tau181 Accuracy in Predicting Core1 Status and Definition of Cut‐Off Value

**FIGURE 2:**
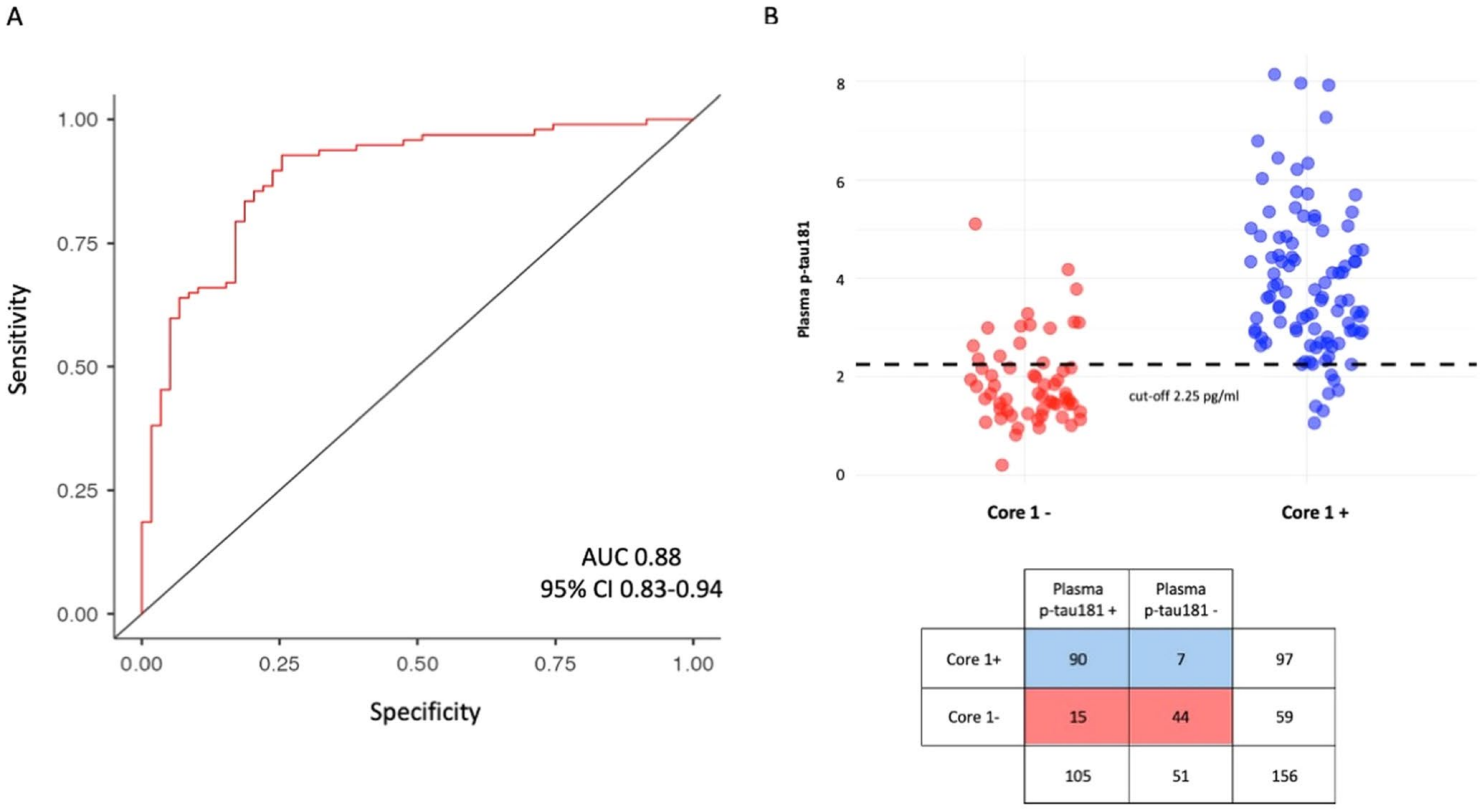
ROC curve analysis of plasma p‐tau181 and single cut‐off approach for classification of Core1+ versus Core1− patients. (A) ROC curve analysis of plasma p‐tau181 in discrimination of Core1+ patients from Core1− patients. (B) Dot plot illustrates the distribution of patients based on plasma p‐tau217 levels categorization according to a single cut approach. The *x*‐axis represents Core1 status, and the *y*‐axis represents plasma p‐tau217 levels. Blue dots: Patients with plasma p‐tau217 levels above the cut‐off. Red dots: Patients with plasma p‐tau217 levels below the cut‐off.

In order to evaluate the diagnostic accuracy of plasma p‐tau181 in distinguishing between Core1+ and Core1− patients, we performed a ROC curve analysis. Plasma p‐tau181 was highly accurate for discriminating between Core1+ and Core1− patients (AUC = 0.88 [95% CI 83.00–94.00]) (Figure 2A). Then, we defined an optimal cut‐off value of 2.25 pg/mL, which discriminated Core1+ from Core1− patients with good accuracy (85.90% [95% CI 80.44–91.36]), sensitivity (92.78% [95% CI 88.72–96.84]), specificity (74.58% [95% CI 67.74–81.41]), PPV (88.18% [95% CI 83.40–92.96]), and NPV (83.09% [95% CI 77.52–88.63]). According to this cut‐off, plasma p‐tau181 levels were dichotomized as positive (p‐tau181 ≥ 2.25 pg/mL) and negative (p‐tau181 < 2.25 pg/mL) (Figure 2B).

### 3.4 Prediction of Conversion to AD Dementia Using Dichotomized Plasma p‐tau181

In a follow up time of 8.85 ± 6.33 years (range 3.00–25.79 years), 13 patients (1 SCD and 12 MCI) converted to AD dementia, while 76 patients (41 SCD and 35 MCI) remained stable. Plasma p‐tau181 levels were significantly higher in converters than in non‐converters (3.63 ± 1.28 vs. 2.25 ± 2.10, *p* < 0.001). In order to determine which factors might influence the risk of conversion to AD dementia, we performed a logistic regression analysis, considering as covariates plasma p‐tau181 levels, age at onset, and *APOE* genotype. The regression model was statistically significant (*χ* ^2^ 19.52, *p <* 0.001). The model explained 39.70% (Nagelkerke *R* ^2^) of the variance and correctly classified 84.5% of cases. Among the covariates, both plasma p‐tau181 levels (OR = 2.20, *p* = 0.040) and *APOE* ε4 genotype (OR = 5.31, *p* = 0.032) were significant predictors of conversion to AD dementia (Table 3).

**TABLE 3:** Logistic regression analysis to predict conversion to AD dementia.

	*B*	*p*	OR	95% CI	
				Lower	Upper
Age at onset	0.05	0.247	1.05	0.96	1.15
Plasma p‐tau181	0.75	**0.040**	2.20	1.03	4.34
*APOE* genotype	1.67	**0.032**	5.31	1.15	24.40

*Note:* Regression Coefficients (*B*), *p* value (*p*), Odds Ratio (OR) and 95% Confidence Intervals (95% CI) for covariates included in the regression models are reported. Significant differences at *p* < 0.05, in **bold characters**.

**FIGURE 3:**
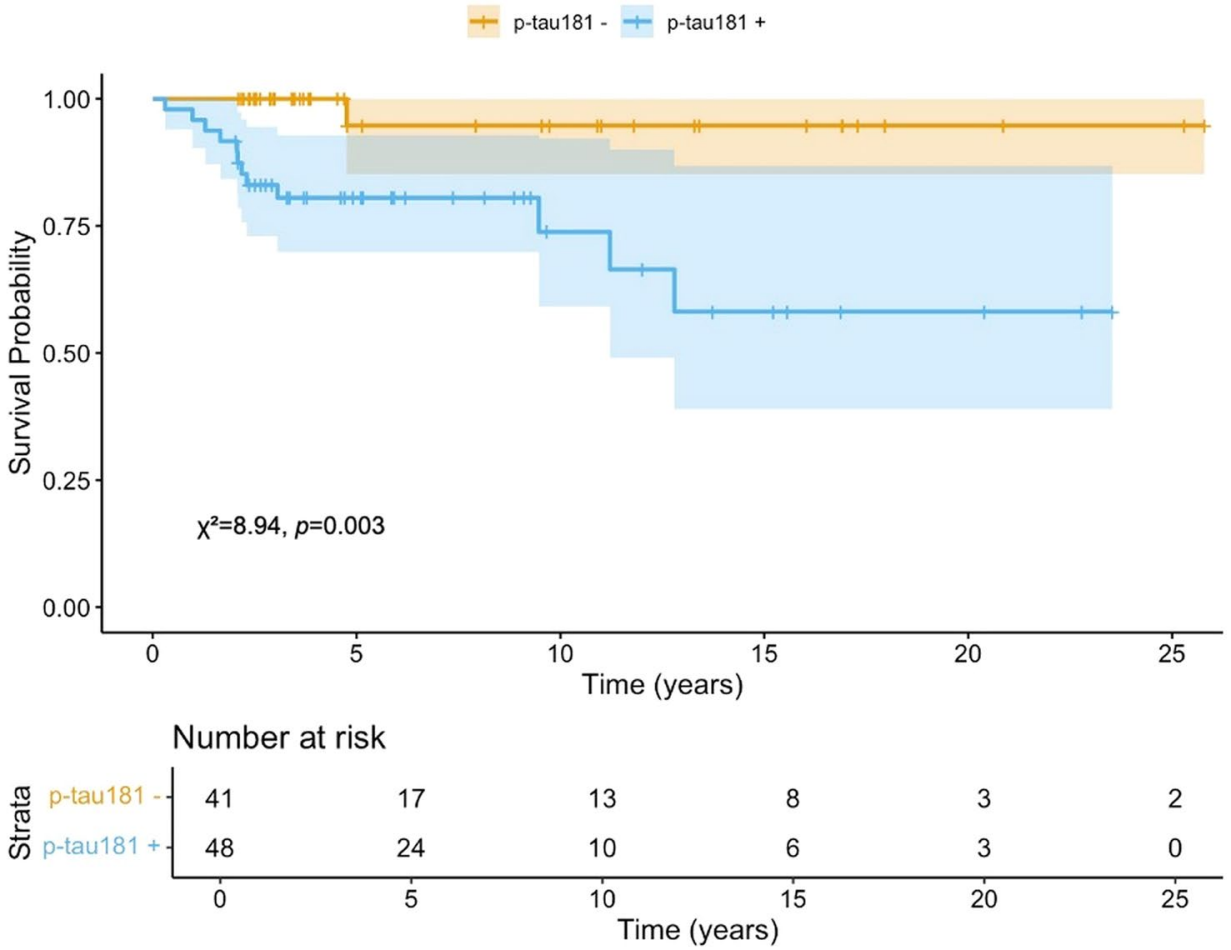
Kaplan–Meier survival analysis comparing conversion to Alzheimer's disease dementia between plasma p‐tau181+ and p‐tau181− patients. For patients who progressed, follow‐up time indicates the time of progression. Number at risk and *p* values for pairwise log‐rank comparisons between groups are reported. Colored shapes indicate 95% confidence interval.

Patients were classified as plasma p‐tau181+ or plasma p‐tau181− according to biomarker levels relative to the defined cut‐off. A higher proportion of conversion to AD dementia was observed in plasma p‐tau181+ patients compared to p‐tau181− patients (92.3% vs. 7.7%, *χ* ^2^ 9.02, *p* = 0.002). The log‐rank test confirmed a significant difference in survival distributions between the two groups (*χ* ^2^ 8.94, *p* = 0.003), supporting the visual separation seen in the Kaplan–Meier curves, with the p‐tau181+ group showing a faster rate of conversion over time (Figure 3). A Cox proportional hazards regression was then conducted to evaluate the association between plasma p‐tau181 status and the risk of conversion to AD dementia. Plasma p‐tau181+ patients showed a significantly increased risk of conversion compared to p‐tau181− patients (HR = 11.65, 95% CI 1.51–89.73, *p* = 0.018). Model discrimination was good, with a concordance index of 0.718.

### 3.5 Longitudinal Change in Cognitive Performance According to Dichotomized Plasma p‐tau181

**FIGURE 4:**
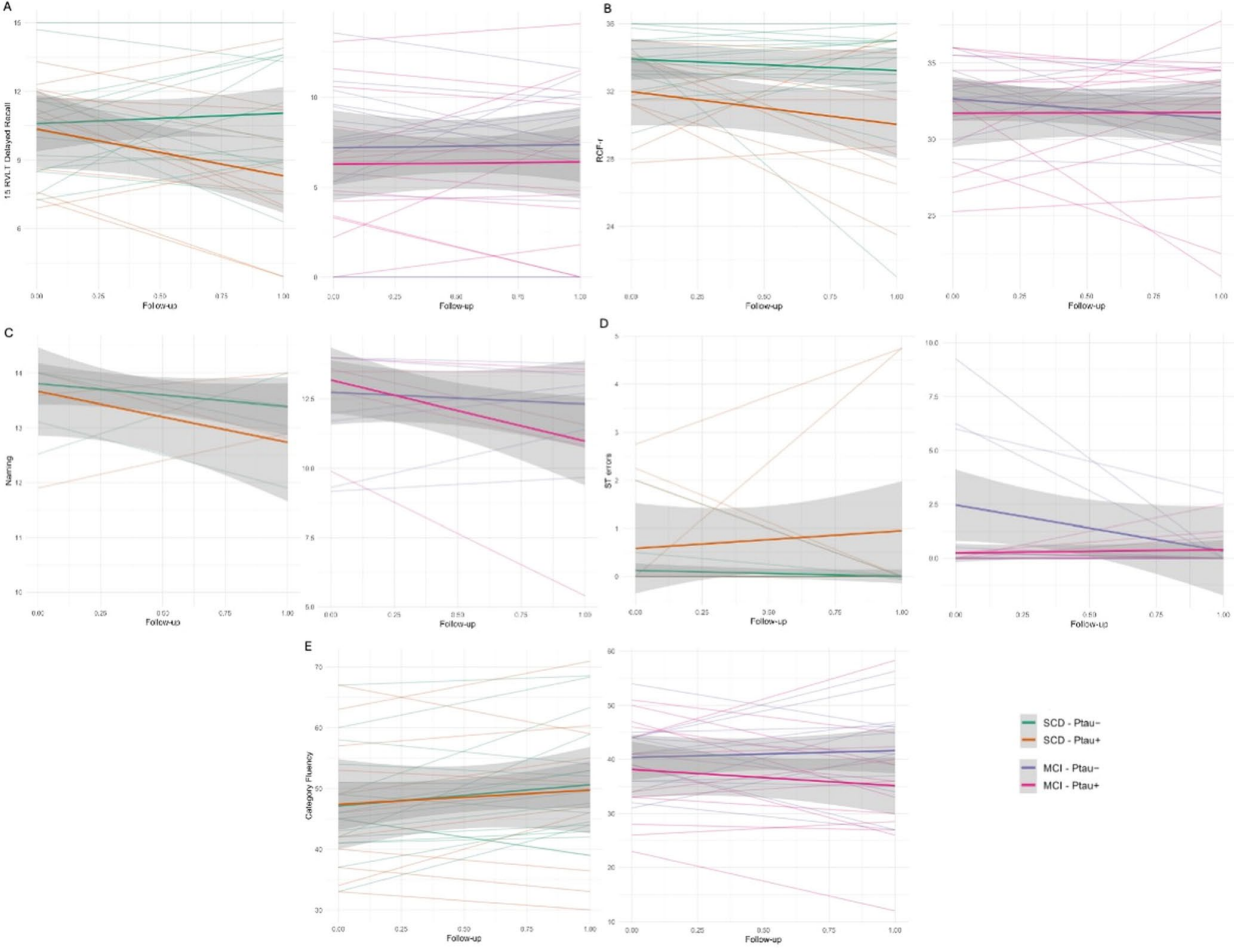
Changes in neuropsychological test scores over time in plasma p‐tau181+ and p‐tau181− patients. Neuropsychological test scores are plotted on the *y*‐axis against follow‐up time on the *x*‐axis. The shaded area represents the 95% confidence interval. Patients are classified according to clinical diagnosis (SCD and MCI) and plasma p‐tau181 status. (A) Changes along time in 15 Rey Auditory Verbal Learning Test (15 RVLT) delayed recall scores. (B) Changes along time in Rey‐Osterrieth complex figure (RCF‐r) delayed recall scores. (C) Changes over time in naming delayed recall scores. (D) Changes over time in Stroop test (ST) number of errors. (E) Changes over time in category fluency test scores.

We examined the longitudinal changes in neuropsychological test scores as a function of diagnostic group (SCD and MCI) and plasma p‐tau181 status (positive or negative) using linear mixed‐effects models (Table S2, Figure 4).

Concerning 15 Rey Verbal Learning Test (15 RVLT) Delayed Recall scores, a significant interaction between time and plasma p‐tau181 status was found (*β* = −4.61, *p* = 0.041), indicating that the change in performance over time differed depending on plasma p‐tau181 status. Post hoc comparisons showed that in the SCD p‐tau181+ patients, scores significantly declined from T0 to T1 (estimate difference = 1.98, *p* = 0.012), while no significant change over time was observed in the other subgroups.

As regard Rey‐Osterrieth complex figure recall (RCF‐r) scores, the model revealed a significant main effect of diagnosis (*β* = −5.39, *p* = 0.007), indicating lower performance in MCI than SCD. An interaction between time and plasma p‐tau181 status was also observed (*β* = −9.36, *p* = 0.010), as well as a significant three‐way interaction among time, diagnosis, and plasma p‐tau181 status (*β* = 4.95, *p* = 0.031). Post hoc comparisons showed a significant decline in performance from T0 to T1in the SCD p‐tau181+ (estimated difference = 3.30, *p* = 0.009).

We also detected a trend to significance in the three‐way interaction between time, clinical diagnosis, and p‐tau181 status (*β* = −2.11, *p* = 0.058) in naming scores, suggesting a potential differential change over time depending on both diagnosis and p‐tau181 status. Indeed, post hoc pairwise comparisons confirmed a significant decline in naming scores from baseline to follow‐up MCI p‐tau181+ (estimated difference = 1.97, *p* < 0.001). No significant changes over time were observed in other subgroups.

Concerning the number of errors at the Stroop test (ST), the model revealed a significant interaction between time and clinical diagnosis (*β* = −2.02, *p* = 0.009), suggesting that the trajectory of Stroop errors over time differed by diagnostic group. Post hoc comparisons demonstrated that only MCI p‐tau181− exhibited a significant reduction in Stroop errors (estimated difference = 2.14, *p* < 0.001).

As regards longitudinal changes on TMT‐A execution time, despite none of the main effects or interaction terms reaching statistical significance, post hoc comparisons showed a significant increase in TMT‐A execution time (estimated difference = −9.58, *p* = 0.039) in SCD p‐tau181−.

Finally, a significant main effect of clinical diagnosis on Category Fluency scores was found (*β* = −6.80, *p* = 0.040), with lower category fluency scores in MCI compared to SCD. No significant main effects of time or plasma p‐tau181 status were detected, nor were any interaction effects. However, post hoc comparisons showed that in the SCD p‐tau181‐ group, category fluency scores significantly increased from T0 to T1 (estimated difference = −3.44, *p* = 0.043).

## 4 Discussion

The Revised Criteria of Alzheimer's Association Workgroup supported the use of plasma p‐tau isoforms for the diagnosis of AD, thus suggesting migrating from more invasive and expensive assays to a more accessible tool [8]. With the recent approval of DMTs, research is focusing on the application of plasma p‐tau species in clinical practice, evaluating both diagnostic and prognostic performances in prodromal and preclinical phases of AD in real world populations [15].

In this context, our study confirmed that plasma p‐tau181 is a reliable biomarker for AD diagnosis, from the earliest phases of cognitive decline, and a good predictor of conversion to AD dementia and worsening in cognitive performance in SCD and MCI patients over time.

First, our results showed that plasma p‐tau181 levels were higher in SCD and MCI patients with AD than in those without, in line with other studies showing higher p‐tau181 levels in Aβ‐positive patients than in Aβ‐negative ones [9, 12]. As expected, the highest levels were found in AD dementia, consistent with previous works showing that plasma p‐tau181 levels increase over time in Aβ‐positive patients, reaching their peak during the dementia stage; moreover, no significant differences were observed between AD‐d and MCI Core1+ patients, highlighting the dynamic nature of this biomarker and its early plateau in the disease course [12, 13].

Considering the recent revision of AD criteria, we evaluated the diagnostic accuracy of plasma p‐tau181 in identifying AD pathology, as defined by Core1 biomarkers. ROC curve analysis revealed that plasma p‐tau181 had good accuracy in distinguishing patients with AD from those without, with an AUC of 0.88. This performance was comparable to previous findings in our cohort for detecting A+T+ patients (classified according to the 2018 ATN system), as well as to results from other studies focusing on the detection of Aβ‐positive individuals [12, 14, 24, 25, 26]. Moreover, we defined a cut‐off of 2.25 pg/mL, which discriminated patients with AD from those without with good accuracy, specificity, and excellent sensitivity. This cut‐off value was slightly lower than the one previously established for detecting AD according to the ATN system in our cohort [14]. This discrepancy might stem from the Revised Criteria, which allow AD to be defined with at least one positive Core1 biomarker, even if the assays themselves specifically measure Aβ (A) or tau (T1) pathophysiology, thus being less restrictive [8].

Interestingly, baseline plasma p‐tau181 levels were predictors of conversion to AD dementia in patients with MCI and SCD. Specifically, individuals with abnormal p‐tau181 concentrations had a markedly increased risk of developing AD dementia, with a hazard ratio of 11.65. These findings align with previous studies; for instance, Janelidze et al. demonstrated that pathological p‐tau181 levels, defined using a specific cut‐off, were associated with a similarly elevated risk of AD dementia, reporting a hazard ratio of 10.90 [9]. Similarly, Tropea et al. showed that MCI with p‐tau181 levels above the defined cut‐off presented a higher risk of developing AD dementia and also a faster functional decline along time [27]. These results have a practical impact: indeed, the prediction of longitudinal progression to AD dementia represents a critical challenge in the clinical management of MCI and SCD. Plasma p‐tau181, like CSF biomarkers of AD, might serve as a highly accurate predictor of the progression to AD dementia in individuals who have not yet developed dementia [28].

Finally, our evaluation of longitudinal changes in neuropsychological scores revealed that baseline plasma p‐tau181 levels were predictive of cognitive worsening in specific domains. Notably, SCD p‐tau181+ patients exhibited significant worsening in long‐term memory‐related tasks, as highlighted by reduced performance in the 15 Rey Verbal Learning Test Delayed Recall and the Rey‐Osterrieth complex figure recall. In contrast, SCD p‐tau181− patients demonstrated a prolonged execution time in the Trail Making Test Part A, whereas MCI p‐tau181− patients showed a reduction in the number of errors on the Stroop test. These findings align with previous studies demonstrating that baseline levels of p‐tau181 can predict cognitive decline across multiple domains [9, 15, 27, 29]. The novel contribution of our study is the identification of cognitive worsening specifically in SCD. Mengel et al. showed that baseline p‐tau181 levels predict a decline in PACC5 scores over time in Aβ‐positive SCD patients. Our work highlights that p‐tau181 can predict progressive decline in memory functions—the earliest affected domain in the amnestic AD—within the SCD population. Therefore, this predictive relationship might be strongly indicative of underlying AD pathology. Conversely, the observed changes in executive function tests among p‐tau181‐negative SCD and MCI patients may reflect different underlying etiologies typical of non‐AD conditions, which tend to affect other cognitive domains [2, 5, 30].

Our study has limitations. Renal function was assessed only dichotomously (presence or absence of renal failure) rather than as a continuous variable like creatinine levels, which future studies should consider for a more accurate evaluation of renal dysfunction's impact on plasma biomarkers [31]. Additionally, the absence of cognitively unimpaired controls and the small sample size may limit generalizability. Finally, the lack of longitudinal plasma p‐tau181 measurements, which could provide insights into test–retest reliability and the potential prognostic value of changes over time. Despite these limitations, the study highlights the diagnostic and prognostic value of plasma p‐tau181, focusing on MCI and SCD—an earlier stage of cognitive decline critical for identifying individuals at risk of AD dementia. Moreover, this study is also one of the first studies to assess plasma p‐tau181's role in detecting worsening of cognitive performance in a well‐defined SCD cohort.

In conclusion, our work confirms that plasma p‐tau181 is not only a good diagnostic marker for AD pathology, but it also plays a role as a predictor of both conversion to AD dementia and of worsening of cognitive performance since the earliest phase of AD. This finding underscores the importance of prognostic biomarkers in distinguishing patients at higher risk of developing AD dementia, enabling clinicians to intervene at a stage where treatments might be most effective and helping researchers select appropriate candidates for clinical trials aimed at delaying or preventing disease progression.

## Author Contributions

**Giulia Giacomucci:** conceptualization, data curation, formal analysis, investigation, methodology, supervision, validation, writing – original draft, writing – review and editing. **Assunta Ingannato, Chiara Crucitti, Silvia Bagnoli, Elisa Marcantelli, Sonia Padiglioni, Valentina Moschini, Carmen Morinelli, Laura Falsini, and Valentina Berti:** data curation, validation, visualization. **Sandro Sorbi:** supervision, validation, visualization. **Benedetta Nacmias:** resources, supervision, validation, visualization. **Valentina Bessi:** project administration, funding acquisition, resources, supervision, validation, visualization, writing – review and editing.

## Ethics Statement

Study procedures and data analysis were performed in accordance with the Declaration of Helsinki and with the ethical standards of the Committee on Human Experimentation of our Institute. The study was approved by the local Institutional Review Board (reference 15691oss). All individuals involved in this research agreed to participate and agreed to have details and results of the research about them published.

## Conflicts of Interest

The authors declare no conflicts of interest.

## Supporting information


**Table S1:** Percentage of positive Alzheimer's disease biomarkers in subjective cognitive decline, mild cognitive impairment and Alzheimer's disease dementia.
**Table S2:** Longitudinal changes in neuropsychological test scores from T0 to T1 in SCD and MCI according to plasma p‐tau181 status.

## Data Availability

All study data, including raw and analyzed data, and materials that support the findings of this study are available from the corresponding author (B.N.) upon reasonable request.
