# Supplementary material for: Plasma p‐tau181 as a Marker of Conversion to Alzheimer's Disease Dementia and Worsening in Cognitive Functions in Subjective Cognitive Decline and Mild Cognitive Impairment: A Longitudinal Study
Source: Ann Clin Transl Neurol. 2025 Sep 10;12(12):2495–504. doi: 10.1002/acn3.70190 (PMC12698954; doi:10.1002/acn3.70190)
Supplement: Supplementary file 1 — Table S1: Percentage of positive Alzheimer's disease biomarkers in subjective cognitive decline, mild cognitive impairment and Alzheimer's disease dementia. Table S2: Longitudinal changes in neuropsychological test scores from T0 to T1 in SCD and MCI according to plasma p‐tau181 status. [file ACN3-12-2495-s001.docx]

**Supplementary Materials**

*Supplementary Table 1: Percentage of positive Alzheimer’s Disease biomarkers in Subjective Cognitive Decline, Mild Cognitive Impairment and Alzheimer’s Disease dementia.*

Values are reported as percentages. Statistically significantly different values between the groups are reported as underlined character. AD-d: Alzheimer’s Disease dementia; CSF: cerebrospinal fluid; MCI: Mild Cognitive Impairment; SCD: Subjective Cognitive Decline. ^a^ χ^2^ 20.93, *p*<0.001; ^b^ χ^2^ 14.92, *p*<0.001; ^c^ χ^2^ 38.08, *p*<0.001; ^d^ χ^2^ 27.22, *p*<0.001.

|  | SCD | MCI | AD-d |
| --- | --- | --- | --- |
| CSF Aβ42/Aβ40 positivity | **33.33% ^a^** | **50.00% ^b^** | **86.04% ^a,b^** |
| CSF p-tau181/Aβ42 positivity | **33.33% ^c^** | **51.51% ^d^** | **100% ^c,d^** |
| Amyloid PET positivity | 33.33% | 57.14% | 100% |

*Supplementary Table 2****:*** *Longitudinal changes in neuropsychological test scores from T0 to T1 in SCD and MCI according to plasma p-tau181 status*

|  |  | **p-tau181 –**  **SCD** | |  | |  |  | **p-tau181 +**  **SCD** | |  | |  | |
| --- | --- | --- | --- | --- | --- | --- | --- | --- | --- | --- | --- | --- | --- |
|  | Mean ± SD | estimate | | *t ratio* | | *p* | Mean ± SD | estimate | | *t ratio* | | *p* | |
| MMSE T0  MMSE T1 | 27.10 ± 2.04  27.64 ± 1.52 | -0.94 | | -1.62 | | 0.109 | 27.85 ± 2.10  26.92 ± 2.03 | 0.59 | | 0.78 | | 0.435 | |
| 15 RVLT-I T0  15 RVLT-I T1 | 51.01 ± 8.33  49.28 ± 11.06 | 3.57 | | 1.78 | | 0.080 | 45.45 ± 9.99  42.21 ± 10.36 | 3.92 | | 1.51 | | 0.135 | |
| 15 RVLT-D T0  15 RVLT-D T1 | 10.66 ± 2.08  11.02 ± 2.62 | -0.35 | | -0.55 | | 0.580 | 10.54 ± 2.16  8.30 ± 3.02 | 1.98 | | 2.58 | | **0.012** | |
| BS-I T0  BS-I T1 | 12.70 ± 4.81  13.00 ± 3.97 | 1.05 | | 1.06 | | 0.297 | 10.10 ± 3. 21  9.70 ± 3.74 | 1.50 | | 1.06 | | 0.292 | |
| BS-D T0  BS-D T1 | 15.96 ± 4.81  16.66 ± 4.16 | 0.61 | | 0.62 | | 0.536 | 13.42 ± 4.45  12.30 ± 2.86 | 2.10 | | 1.50 | | 0.141 | |
| TMT-A T0  TMT-A T1 | 27.65 ± 12.21  38.00 ± 16.04 | -9.58 | | -2.11 | | **0.038** | 32.57 ± 17.37  32.00 ± 19.35 | -4.20 | | -0.67 | | 0.501 | |
| TMT-B T0  TMT-B T1 | 61.61 ± 46.30  85.20 ± 50.60 | -19.50 | | -1.34 | | 0.187 | 73.88 ± 45.27  80.80 ± 45.48 | -14.70 | | -0.68 | | 0.498 | |
| ViS T0  ViS T1 | 48.27 ± 6.55  47.80 ± 6.12 | -0.78 | | -0.47 | | 0.637 | 46.77 ± 7.29  42.69 ± 11.28 | 2.75 | | 1.35 | | 0.181 | |
| FAB T0  FAB T1 | 17.93 ± 1.25  16.43 ± 0.93 | 0.82 | | 1.02 | | 0.309 | 16.17± 1.48  15.61 ± 2.03 | 0.83 | | 0.88 | | 0.381 | |
| VS-F T0  VS-F T1 | 6.18 ± 0.94  5.93 ± 1.03 | 0.28 | | 1.10 | | 0.278 | 6.05 ± 1.10  5.85 ± 0.70 | -0.12 | | -0.33 | | 0.739 | |
| VS-B T0  VS-B T1 | 4.51 ± 1.01  4.07 ± 1.19 | 0.46 | | 1.81 | | 0.076 | 4.14 ± 1.14  4.02 ± 0.93 | -0.31 | | -0.89 | | 0.378 | |
| SS-F T0  SS-F T1 | 5.03 ± 0.92  5.05 ± 0.76 | 0.08 | | 0.37 | | 0.713 | 5.13 ± 0.89  5.23 ± 0.72 | -0.33 | | -1.09 | | 0.282 | |
| SS-B T0  SS-B T1 | 4.52 ± 0.96  4.71 ± 0.75 | -0.08 | | -0.31 | | 0.757 | 4.66 ± 1.17  4.68 ± 0.70 | -0.27 | | -0.74 | | 0.460 | |
| RFC-c T0  RFC-c T1 | 34.51 ± 4.23  33.91 ± 1.71 | 0.65 | | 0.68 | | 0.499 | 32.88 ± 2.83  30.04 ± 3.82 | 1.88 | | 1.53 | | 0.131 | |
| RCF-r T0  RCF-r T1 | 21.75 ± 5.35  23.45 ± 5.51 | -1.11 | | -1.17 | | 0.245 | 19.64 ± 4.12  16.76 ± 6.51 | 3.30 | | 2.69 | | **0.009** | |
| SCW-T T0  SCW-T T1 | 14.09 ± 7.08  12.59 ± 8.29 | 1.11 | | 0.33 | | 0.736 | 17.86 ± 12.36  20.57 ± 16.14 | -3.04 | | -0.64 | | 0.524 | |
| SCW-E T0  SCW-E T1 | 0.19 ± 0.48  0.00 ± 0.00 | 0.12 | | 0.29 | | 0.770 | 0.41± 0.89  4.75 ± 0.00 | -0.33 | | -0.57 | | 0.569 | |
| PFT T0  PFT T1 | 37.95 ± 10.35  40.69 ± 10.27 | -1.94 | | -0.87 | | 0.385 | 40.34 ± 12.08  44.62 ± 14.48 | -3.70 | | -1.32 | | 0.192 | |
| CFT T0  CFT T1 | 44.52 ± 12.00  50.59 ± 8.75 | -3.44 | | -2.07 | | **0.04** | 47.30 ± 10.03  49.71 ± 12.11 | -1.82 | | -0.83 | | 0.405 | |
| NA T0  NA T1 | 13.75 ± 0.48  13.08 ± 0.83 | 0.34 | | 0.62 | | 0.540 | 13.77 ± 2.76  12.73 ± 1.65 | 0.46 | | 0.73 | | 0.469 | |
|  |  | **p-tau181 -**  **MCI** | |  | |  |  | **p-tau181 +**  **MCI** | |  | |  | |
|  | Mean ± SD | estimate | | *t ratio* | | *p* | Mean ± SD | estimate | | *t ratio* | | *p* | |
| MMSE T0  MMSE T1 | | 27.04 ± 2.39  26.87 ± 2.49 | -0.70 | -1.01 | | 0.31 | | 25.79 ± 2.24  24.91 ± 3.52 | 0.56 | 0.81 | | 0.403 | |
| 15 RVLT-I T0  15 RVLT-I T1 | | 38.49 ± 9.72  38.07 ± 9.51 | 0.95 | 0.39 | | 0.693 | | 37.00 ± 9.27  36.01 ± 11.39 | 0.46 | 0.19 | | 0.843 | |
| 15 RVLT-D T0  15 RVLT-D T1 | | 7.13 ± 3.63  8.60 ± 2.11 | -0.18 | -0.26 | | 0.795 | | 6.49 ± 3.90  7.15 ± 3.15 | -0.12 | -0.18 | | 0.856 | |
| BS-I T0  BS-I T1 | | 8.88 ± 3.55  8.55 ± 3.74 | 2.50 | 1.59 | |  | | 6.88 ± 3.87  6.57 ± 2.99 | -0.33 | -0.18 | | .855 | |
| BS-D T0  BS-D T1 | | 10.23 ± 4.08  8.87 ± 4.15 | 1.87 | 1.19 | | 0.238 | | 8.24 ± 4.82  10.23 ± 7.48 | -3.27 | -1.81 | | 0.07 | |
| TMT-A T0  TMT-A T1 | | 44.74 ± 27.43  39.00 ± 17.19 | -6.71 | -1.26 | | 0.212 | | 43.68 ± 26.06  44.25 ± 22.26 | -8.83 | -1.71 | | 0.09 | |
| TMT-B T0  TMT-B T1 | | 123.38 ± 70.92  109.17 ± 45.45 | 15.7 | 0.88 | | 0.383 | | 127.39 ± 139.13  139.75 ± 79.06 | -14.0 | -0.65 | | 0.517 | |
| ViS T0  ViS T1 | | 42.32 ± 9.71  40.00 ± 9.76 | -1.07 | -0.56 | | 0.571 | | 38.25 ± 11.09  35.04 ± 10.37 | 0.63 | 0.34 | | 0.735 | |
| FAB T0  FAB T1 | | 15.52 ± 1.89  17.52 ± 6.06 | -1.45 | -1.57 | | 0.122 | | 14.60 ± 2.54  14.06 ± 2.68 | -0.15 | -0.15 | | 0.874 | |
| VS-F T0  VS-F T1 | | 5.88 ± 1.02  5.29 ± 0.89 | 0.56 | 1.44 | | 0.157 | | 5.98 ± 0.84  6.40 ± 0.68 | -0.07 | -0.159 | | 0.874 | |
| VS-B T0  VS-B T1 | | 3.86 ± 0.77  4.18 ± 1.41 | -0.43 | -1.10 | | 0.274 | | 4.00 ± 1.04  4.18 ± 0.96 | 0.25 | 0.54 | | 0.589 | |
| SS-F T0  SS-F T1 | | 4.93 ± 0.87  4.94 ± 1.14 | -0.07 | -0.22 | | 0.821 | | 5.09 ± 1.02  5.25 ± 1.13 | 0.59 | 1.48 | | 0.148 | |
| SS-B T0  SS-B T1 | | 4.28 ± 0.91  4.50 ± 0.88 | -0.27 | -0.69 | | 0.492 | | 4.17 ± 0.98  4.56 ± 1.15 | 0.09 | 0.19 | | 0.843 | |
| RFC-c T0  RFC-c T1 | | 31.65 ± 3.12  31.67 ± 2.60 | 1.35 | 1.19 | | 0.238 | | 31.77 ± 3.49  31.75 ± 4.78 | -0.05 | -0.04 | | 0.962 | |
| RCF-r T0  RCF-r T1 | | 15.35 ± 5.28  17.11 ± 5.98 | 0.47 | 0.40 | | 0.686 | | 12.66 ± 5.64  13.94 ± 5.74 | -0.05 | -0.04 | | 0.963 | |
| ST-t T0  ST-t T1 | | 28.14 ± 20.37  23.50 ± 15.92 | 7.28 | 1.54 | | 0.128 | | 26.75 ± 18.37  28.93 ± 19.15 | -3.72 | -0.86 | | 0.392 | |
| ST-e T0  ST-e T1 | | 1.71 ± 3.15  1.50 ± 2.12 | 2.14 | 3.60 | | **0.007** | | 2.45 ± 5.33  0.79 ± 1.00 | -0.20 | -0.36 | | 0.714 | |
| PFT T0  PFT T1 | | 34.43 ± 11.47  35.87 ± 10.18 | 0.91 | 0.35 | | 0.726 | | 34.86 ± 11.60  30.41 ± 10.96 | -0.12 | -0.04 | | 0.961 | |
| CFT T0  CFT T1 | | 41.25 ± 8.55  41.61 ± 8.86 | -1.26 | -0.65 | | 0.519 | | 38.13 ± 9.02  35.14 ± 10.74 | 3.10 | 1.60 | | 0.114 | |
| NA T0  NA T1 | | 12.02 ± 3.61  12.32 ± 1.52 | -0.25 | 0.62 | | 0.607 | | 12.30 ± 2.16  10.60 ± 3.01 | 1.96 | 3.89 | | **0.008** | |

MMSE *Mini Mental State Evaluation*; 15-RVLT-I *Rey auditory Verbal Learning test immediate recall*; 15-RVLT-D *Rey auditory Verbal Learning test delayed recall*; BS-I *Babcock Short Story immediate recall*; BS-D *Babcock Short Story delayed recall*; TMT-A *Trail Making Test part A*; TMT-B *Trail Making Test part B*; ViS *Visual Search;* FAB *Frontal Assessment Battery*; VS-F *Verbal Span forward*; VS-B *Verbal Span backward;* SSF *Spatial Span forward*; SS-B *Spatial Span backward*; RCF-c *Rey-Osterrieth Complex Figure copy*; RCF-r *Rey-Osterrieth Complex Figure delayed* *recall*; ST-t *Stroop Test execution time*; ST-e *Stroop Test number of errors*; PFT *Phonemic Fluency Test*; CFT *Category* *Fluency Test*; NA *Naming Test*.
